# Structured reporting of metastatic disease for improving communication in comprehensive cancer centers- a feasibility study

**DOI:** 10.1186/1470-7330-15-S1-P30

**Published:** 2015-10-02

**Authors:** J Tesdorff, D Simons, H-P Schlemmer

**Affiliations:** 1 German Cancer Research Center Heidelberg, Department of Radiology, Germany

## Background

To communicate imaging findings and interpretation precisely and comprehensively within a multidisciplinary treatment team written radiology reports are essential. Structured reporting systems increasingly take advantage of conventional free-form reports. Therefore we developed a visual imaging report chart and evaluated its acceptance by radiologists and oncologists in comprehensive cancer centers.

## Method

Six imaging report charts of representative CT follow-up examination of patients with metastatic melanoma were recorded. Each chart includes a short and focused written text as well as a visual reporting diagram, which is composed of two sections: (1) patient characteristics; and (2) schematic documentation and graphical and pictorial visualization of the radiologic findings including documentation of imaging findings, treatment response according to qualitative or standardised criteria, clinical recommendation and the course of tumour burden. In total 36 reviewers (18 radiologists, 18 clinicians) compared the report charts with the corresponding conventional free-text report by a questionnaire grading their degree of satisfaction by a 10 point Likert-scale. Statistical significance was evaluated by one-way analysis of variance (ANOVA) and Student’s t-test.

## Results

Referring oncologists rated improvement of therapy decision making for oncologic patients significantly higher than radiologists (p<0.01). A significant difference (p<0.005) was observed between radiologists and clinicians regarding their opinion on time-saving in working routine due to our reporting chart (5.1 vs. 8.7 respectively).

## Conclusion

Structured reporting and documentation of CT examinations in metastatic disease is well appreciated by referring oncologists. The proposed standardised imaging report chart has the potential to improve therapy decision making in the multidisciplinary context.

